# Supplementary material for: Amphetamine in adolescence induces a sex-specific mesolimbic dopamine phenotype in the adult prefrontal cortex
Source: Commun Biol. 2025 Dec 6;9:12. doi: 10.1038/s42003-025-09239-6 (PMC12770397; doi:10.1038/s42003-025-09239-6)
Supplement: Supplementary file 1 — Supplementary Fig. 1 [file 42003_2025_9239_MOESM1_ESM.pdf]

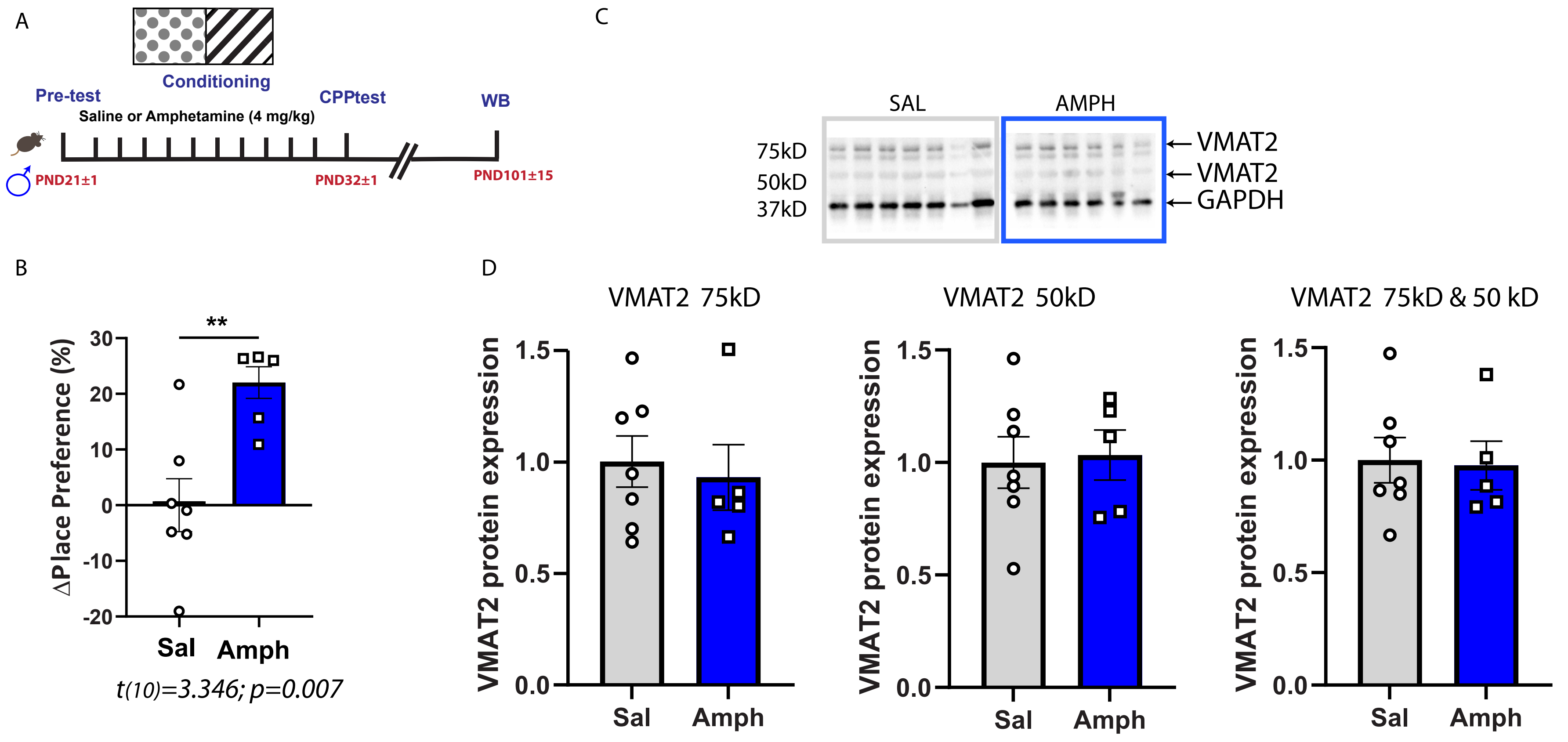

Supplementary Figure 1. AMPH in adolescence does not alter VMAT2 levels in the adult PFC of male mice.
A. Experimental timeline. **B.** Male mice exposed to AMPH (4.0 mg/kg) in early adolescence developed place preference for the side of the box paired with the drug. **C.** Western blot image from adult male mice (PND101±15) exposed to saline or AMPH in adolescence (PND21). The two isoforms for vesicular monoamine transporter 2 (VMAT-2; 56kDa and 70kDa) and GAPDH reference band (~37kDa) are indicated. **D.** There is no difference in the expression of VMAT-2 in the PFC of mice that were exposed to AMPH in adolescence compared to their saline counterparts. All bar graphs are presented as mean values ±SEM. Saline n=7; Amphetamine n= 5. Descriptive statistics and statistical tests performed are described in detail in the Supplementary Data 1. The underlying source data for the graphs is presented in Supplementary Data 2.
