# Supplementary material for: Amphetamine in adolescence induces a sex-specific mesolimbic dopamine phenotype in the adult prefrontal cortex
Source: Commun Biol. 2025 Dec 6;9:12. doi: 10.1038/s42003-025-09239-6 (PMC12770397; doi:10.1038/s42003-025-09239-6)
Supplement: Supplementary file 2 — Description of Additional Supplementary files [file 42003_2025_9239_MOESM2_ESM.docx]

**Description of Additional Supplementary files**

1. File name: Supplementary Figure 1
2. Description: Supplementary Figure 1 showing VMAT2 levels in adulthood of animals treated with AMPH in adolescence.
3. File name: Supplementary data 1.
4. Description: Descriptive statistics and statistical analyses conducted for the data presented in Figures 1 through 5 and Supplementary Figure 1.
5. File name: Supplementary data2.
6. Description: Underlying source data for the graphs is presented in Figures 1 through 5 and Supplementary Figure 1
