# Supplementary material for: Amphetamine in adolescence induces a sex-specific mesolimbic dopamine phenotype in the adult prefrontal cortex
Source: Commun Biol. 2025 Dec 6;9:12. doi: 10.1038/s42003-025-09239-6 (PMC12770397; doi:10.1038/s42003-025-09239-6)
Supplement: Supplementary file 5 — Reporting Summary [file 42003_2025_9239_MOESM5_ESM.pdf]

Corresponding author(s): Cecilia Flores

Last updated by author(s): Oct 2, 2025

Reporting Summary

Nature Portfolio wishes to improve the reproducibility of the work that we publish. This form provides structure for consistency and transparency in reporting. For further information on Nature Portfolio policies, see our Editorial Policies and the Editorial Policy Checklist.

Statistics

For all statistical analyses, confirm that the following items are present in the figure legend, table legend, main text, or Methods section.

n/a	Confirmed	
☐	☒	The exact sample size (n) for each experimental group/condition, given as a discrete number and unit of measurement
☒	☐	A statement on whether measurements were taken from distinct samples or whether the same sample was measured repeatedly
☐	☒	The statistical test(s) used AND whether they are one- or two-sided<br>*Only common tests should be described solely by name; describe more complex techniques in the Methods section.*
☒	☐	A description of all covariates tested
☐	☒	A description of any assumptions or corrections, such as tests of normality and adjustment for multiple comparisons
☐	☒	A full description of the statistical parameters including central tendency (e.g. means) or other basic estimates (e.g. regression coefficient) AND variation (e.g. standard deviation) or associated estimates of uncertainty (e.g. confidence intervals)
☐	☒	For null hypothesis testing, the test statistic (e.g. F, t, r) with confidence intervals, effect sizes, degrees of freedom and P value noted<br>*Give P values as exact values whenever suitable.*
☒	☐	For Bayesian analysis, information on the choice of priors and Markov chain Monte Carlo settings
☒	☐	For hierarchical and complex designs, identification of the appropriate level for tests and full reporting of outcomes
☐	☒	Estimates of effect sizes (e.g. Cohen's d, Pearson's r), indicating how they were calculated

Our web collection on statistics for biologists contains articles on many of the points above.

Software and code

Policy information about availability of computer code

Data collection	Stereoinvestigator 2021.1.1, Neurolucida 2022.1.1.Toxtrac, TDT Synapse
Data analysis	GraphPad Prism 10, SPSS 28, Matlab 2023a Code was submitted along the paper and is available in figshare

For manuscripts utilizing custom algorithms or software that are central to the research but not yet described in published literature, software must be made available to editors and reviewers. We strongly encourage code deposition in a community repository (e.g. GitHub). See the Nature Portfolio guidelines for submitting code & software for further information.

Data

Policy information about availability of data

All manuscripts must include a data availability statement. This statement should provide the following information, where applicable:

- Accession codes, unique identifiers, or web links for publicly available datasets
- A description of any restrictions on data availability
- For clinical datasets or third party data, please ensure that the statement adheres to our policy

The source data was provided with this paper and the data supporting the finding and the MATLAB code are openly available in figshare
https://doi.org/10.6084/m9.figshare.30267643.v1
https://doi.org/10.6084/m9.figshare.30267817.v1

Research involving human participants, their data, or biological material

Policy information about studies with human participants or human data. See also policy information about sex, gender (identity/presentation), and sexual orientation and race, ethnicity and racism.

Reporting on sex and gender	N/A
Reporting on race, ethnicity, or other socially relevant groupings	N/A
Population characteristics	N/A
Recruitment	N/A
Ethics oversight	N/A

Note that full information on the approval of the study protocol must also be provided in the manuscript.

Field-specific reporting

Please select the one below that is the best fit for your research. If you are not sure, read the appropriate sections before making your selection.

☒ Life sciences ☐ Behavioural & social sciences ☐ Ecological, evolutionary & environmental sciences

For a reference copy of the document with all sections, see nature.com/documents/nr-reporting-summary-flat.pdf

Life sciences study design

All studies must disclose on these points even when the disclosure is negative.

Sample size	No sample size was calculated a priori. Suitable sample sizes were estimated based on previous experiments (e.g. Reynolds et al 2018, 2023)
Data exclusions	Fiber photometry experiment: One male mouse from the saline group and one male mouse from the amphetamine group, both exposed during adolescence, were excluded due to insufficient signal. Among the remaining subjects, an additional male mouse from the adolescent saline group did not exhibit a distinct saline peak and was therefore omitted from this portion of the analysis. For female mice, data could not be obtained from two individuals treated with amphetamine and two treated with saline in adolescence, owing to either lack of signal or loss of the headcap containing the optical fiber. Regarding animals subjected to CRISPRa, two mice from the LacZ group one that received saline and one that received amphetamine during adolescence did not show fluorescent signal. One individual from the Dcc overexpression group, that received AMPH during adolescence was excluded as an outlier from the analysis.<br><br>Western Blot :One of the samples obtained from the amphetamine treated group was removed from the analysis since it was an outlier.<br>Neuroanatomical Anlaysis: Two male subjects who received amphetamine were excluded from the stereological analysis because their coronal brain sections were damaged during processing and handling for immunofluorescent and DAT labeling, making them unsuitable for stereological analysis.
Replication	All experiments were replicated with success meaning that group measures (mean, SD) did not vary significantly across experimental cohorts (minimum 2) tested on separate occasions.
Randomization	Mice were randomly assigned to experimental groups, with counterbalancing in consideration for minimizing litter/cagemate effects
Blinding	Experimenters were blinded to group allocation during neuroanatomical analysis

Reporting for specific materials, systems and methods

We require information from authors about some types of materials, experimental systems and methods used in many studies. Here, indicate whether each material, system or method listed is relevant to your study. If you are not sure if a list item applies to your research, read the appropriate section before selecting a response.

Materials & experimental systems

n/a	Involved in the study
☐	☒ Antibodies
☐	☒ Eukaryotic cell lines
☒	☐ Palaeontology and archaeology
☐	☒ Animals and other organisms
☒	☐ Clinical data
☒	☐ Dual use research of concern
☒	☐ Plants

Methods

n/a	Involved in the study
☒	☐ ChIP-seq
☒	☐ Flow cytometry
☒	☐ MRI-based neuroimaging

Antibodies

Antibodies used

Monoclonal rat anti-dopamine transporter (DAT) antibody(Millipore Sigma MAB369), Polyclonal rabbit anti-tyrosinehydroxylase (TH) antibody (Millipore Sigma AB152), Monoclonal Mouse anti-TH (Millipore Sigma MAB318), polyclonal rabbit anti-VMAT2,Rabbit antired flourescent protein (RFP) for mCherry antibody (RocLAND 600-401-379), Chicken anti-GFP antibody (Abcam ab13970) Goat anti-Rat Alexa Fluor 488 (invitrogen, A-1106), Donkey anti-Rabbit Alexa Fluor 594-conjugated (invitrogen, A-21207),Goat anti-mouse Alexa Fluor 488 antibody (invitrogen A-11001) Chicken anti-GFP antibody (abcam ab13970),Alexa-488-conjugated goat-anti-chicken secondary antibody (ATT-Bio, 166687)

Validation

Polyclonal rabbit anti TH antibody AB152
https://www.sigmaaldrich.com/CA/en/product/mm/ab152

Monoclonal mouse anti TH antibody (Millipore Sigma Cat No. MAB 318)
https://www.sigmaaldrich.com/CA/en/product/mm/mab318

Monoclonal rat anti-dopamine transporter (DAT) antibody (Millipore sigma MAB369)
https://www.sigmaaldrich.com/CA/en/product/mm/mab369

Polyclonal rabbit anti-VMAT2
https://pubmed.ncbi.nlm.nih.gov/27836486/

Rockland Rabbit antiRFP
https://www.rockland.com/categories/primary-antibodies/rfp-antibody-pre-adsorbed-600-401-379/?srsltid=AfmBOoorj9MjZ-BAzKcPK0A8LulqZdfIDDz3o331Vrl89G-5gRzbSVYQ

AF conjugated secondaries
https://www.thermofisher.com/ca/en/home/life-science/antibodies/secondary-antibodies/fluorescent-secondary-antibodies/alexa-fluor-secondary-antibodies.html

In addition, all antibodies have been previously validated in publications from our or our collaborators laboratories

Eukaryotic cell lines

Policy information about cell lines and Sex and Gender in Research

Cell line source(s)	N/A
Authentication	No procedure
Mycoplasma contamination	Cell lines were not tested for contamination
Commonly misidentified lines (See ICLAC register)	No commonly misidentified cell lines were used

Animals and other research organisms

Policy information about studies involving animals; ARRIVE guidelines recommended for reporting animal research, and Sex and Gender in Research

Laboratory animals	C57BL/6J mice were used starting from PND21
Wild animals	N/A
Reporting on sex	Sex was considered in the study design and most experiments were conducted in both sexes with comparisons were applicable
Field-collected samples	N/A

Ethics oversight	Canadian Council of Animal Care and the McGill University/Douglas Mental Health University Institute Animal Care Committee

Note that full information on the approval of the study protocol must also be provided in the manuscript.

Plants

Seed stocks	N/A
Novel plant genotypes	N/A
Authentication	N/A
